# Timing of dietary zinc additions during gestation for improved piglet survival

**DOI:** 10.1093/tas/txae030

**Published:** 2024-03-08

**Authors:** Kelsey L Hammers, Pedro E Urriola, Mark Schwartz, Moon-Suhn Ryu, Andres Gomez, Lee J Johnston

**Affiliations:** Department of Animal Science, University of Minnesota, St. Paul, MN 55108, USA; Department of Animal Science, University of Minnesota, St. Paul, MN 55108, USA; Schwartz Farms Inc., SleepyEye, MN 56085, USA; Department of Food and Nutrition, Yonsei University, Seoul, South Korea; Department of Animal Science, University of Minnesota, St. Paul, MN 55108, USA; Department of Animal Science, University of Minnesota, St. Paul, MN 55108, USA; West Central Research and Outreach Center, University of Minnesota, Morris, MN 56267, USA

**Keywords:** mortality, preweaning, swine, zinc

## Abstract

The objectives of this study were to determine a practical approach to feeding elevated dietary zinc (Zn) to gestating sows in a commercial setting and to confirm preweaning mortality could be reduced by feeding high Zn to sows during different periods of gestation. The study was conducted at a commercial sow farm in the upper Midwest. Mixed parity sows (*n* = 267) over three consecutive weekly farrowing groups (sows farrowing within 1 wk) were assigned randomly to one of the three dietary treatments within parity. Treatments consisted of: (1) control sows fed a corn–soybean meal diet containing 206 mg/kg total supplemental Zn supplied by zinc hydroxychloride; (2) breed-to-farrow: as control + 147 mg/kg supplemental Zn as ZnSO_4_ (353 mg/kg total supplemental Zn) fed from 5 d after breeding to farrowing; and (3) day 110-to-farrow: as control fed from breeding to farrowing + 4,079 mg/kg supplemental Zn as ZnSO_4_ (4,285 mg/kg total supplemental Zn) starting day 110 of gestation until farrowing. At farrowing, individual piglets were weighed and identified within 12 h of birth. Data were analyzed using PROC GLIMMIX of SAS and the model considered the fixed effect of dietary treatment and random effect of farrowing group. Dietary treatments did not affect number of total pigs born per litter. For breed-to-farrow sows, there was an increase in the percentage of pigs born alive compared to sows fed the control and day 110-to-farrow treatments (*P* < 0.001). The number of stillborn pigs expressed as a percentage of total litter size at birth decreased for breed-to-farrow sows (*P* < 0.001) compared with control or day 110-to-farrow sows. Mortality of low birth weight piglets from birth to weaning did not differ among dietary treatments (*P *= 0.305); however, a trend for decreasing post-natal mortality (*P *= 0.068) of normal birth weight pigs was observed for pigs born to sows fed elevated Zn 5 d before farrowing. In conclusion, feeding elevated Zn to sows throughout gestation increased the proportion of pigs born alive suggesting that elevated gestational Zn intake makes piglets more robust to endure the stresses of farrowing and decreases intrapartum mortality. Under the conditions of this study, elevated Zn intake of sows did not influence piglet post-natal survival. However, feeding high zinc throughout gestation may decrease piglet mortality during the parturition process.

## Teaser

Supplementing high dietary zinc to gestating sows throughout gestation or 5 d before farrowing did not decrease piglet preweaning mortality. However, intrapartum mortality of piglets decreased when sows consumed high levels of zinc throughout gestation.

## Introduction

Productivity of the swine industry has improved through the use of modern sow genetic lines in recent decades, allowing for an average of 15.4 total pigs born per litter (PigCHAMP, 2022), compared to 13.2 total pigs born per litter a decade ago (PigCHAMP, 2012; Knauer and Hostetler, 2013). While the progression of genetic selection in the swine industry has increased average litter size at birth, the number of pigs weaned per litter has not increased as markedly (PigCHAMP, 2022). This difference can be attributed to a rise in preweaning mortality, exacerbated by large litter sizes with increased variation of piglet birth weight within litters (Milligan et al., 2002; Wolf et al., 2008; Yuan et al., 2015), increased prevalence of pigs born weighing less than 1.0 kg (Quesnel et al., 2008; Han et al., 2021), and the sow’s limited ability to nurse large litters when pigs outnumber functional teats (Edwards and Baxter, 2015). Preweaning mortality of piglets in commercial settings averages about 16% (PigCHAMP, 2022) while low birth weight pigs weighing less than 1.0 kg at birth can experience mortality rates up to 80 percent (Zeng et al., 2019). The exact definition of a low birth-weight pig remains ambiguous. Researchers have set birth weight thresholds ranging from 0.95 to 1.13 kg and defined a low birth weight pig as one that falls below their selected threshold (Bergstrom, 2011; Jourquin et al., 2016; Calderón Díaz et al., 2017; Feldpausch et al., 2019; Zeng et al., 2019; Holen et al., 2020). Low birth weight pigs suffer extremely high mortality rates and have a significant disadvantage competing with heavier littermates for colostrum intake which is essential for post-natal survival (Declerck et al., 2016). Low birth weight pigs have reduced vigor and viability and are unable to regulate body temperature (Herpin et al., 2002; Peltoniemi et al., 2021). Birth weight is correlated positively with survivability (Baxter et al., 2008; Fix et al., 2010). Therefore, improving piglet survivability regardless of birth weight is economically relevant to the swine industry.

Maternal nutrition has lasting effects on the fetus that can affect physiological functions long after birth (Ji et al., 2017). Fetal imprinting methods may provide the fetus with an enhanced ability to survive the post-natal period through increases in birth weight and/or piglet robustness. Supplementation of elevated zinc (Zn; 360 to 595 mg/kg) to gestating sows has increased birth weight (Holen et al., 2020; Hu et al., 2023) and reduced preweaning mortality of low birth weight pigs (Vallet et al., 2014; Holen et al., 2020). High Zn also decreased frequency of stillborn pigs (Vallet et al., 2014) and overall piglet mortality during lactation (Cony et al., 2023). Therefore, high levels of Zn fed to sows may have utility in a fetal imprinting strategy. The NRC (2012) states the Zn requirement of breeding swine is not well established. Thus, the Zn requirement of modern sow genotypes may be higher than values established decades ago by dated studies (Hedges et al., 1976; Kirchgessner et al., 1981) due to the demands of fetal growth, milk synthesis, and tissue repair. Zinc is vital for proper fetal development and post-natal growth among many other biological functions (Ryu and Aydemir, 2020). Zinc is critical for fetal development and accumulates at high concentrations in fetuses during early stages of gestation (Hostetler et al., 2003). Decreased mortality observed in these studies resulted when high dietary Zn supplementation began on days 80 or 85 of gestation through parturition. Changing diet formulation or providing an additional supplement to increase dietary Zn concentration for a portion of gestation is not practical in most commercial settings. Consequently, we selected two supplementation strategies that could be implemented easily and still provide high dietary Zn. Therefore, the objective of this study was to determine a practical approach to feeding elevated dietary Zn to gestating sows in a commercial setting that would reduce preweaning mortality. We hypothesized preweaning mortality could be reduced by feeding elevated levels of Zn to sows during different gestational periods other than from day 85 of gestation to farrowing.

## Materials and Methods

The experimental protocol was reviewed and approved by the University of Minnesota’s Institutional Animal Care and Use Committee (IACUC# 2009-38423A). The experiment began in December 2020 and concluded in May 2021. This experiment was conducted on a commercial sow farm (2,500 sows) in the upper Midwest region of the United States.

### Animals, Housing, and Treatments

Three consecutive weekly farrowing groups (cohorts of sows that farrowed within 1 wk) that included 267 females (parity 0 to 6; PIC Camborough, Hendersonville, TN) were assigned randomly to one of the three dietary treatments within parity about 5 d post-breeding.

Dietary treatments were formulated to consist of: (1) control—sows fed a corn–soybean meal-based diet containing 206 mg/kg total supplemental zinc supplied by zinc hydroxychloride (Intellibond Z, Micronutrients, Indianapolis, IN); (2) breed-to-farrow—as control + 147 mg/kg supplemental Zn as ZnSO_4_ fed from 5 d post-breeding to farrowing; and (3) day 110-to-farrow—as control + 4,079 mg/kg supplemental Zn as ZnSO_4_ starting on day 110 of gestation until farrowing. Supplemental Zn concentrations of the control, breed-to-farrow, and day 110-to-farrow dietary treatments were designed to be 125 mg/kg, 266 mg/kg, and 2,840 mg/kg, respectively. However, actual intakes of supplemental Zn were higher than designed based on analyzed Zn concentration of top-dresses and gestation and lactation diets (see below). The composition of gestation and lactation diets was based on the farm’s standard operating procedures (Table 1). Dietary treatments were imposed by adding 60 mL (45 g; breed-to-Ffarrow) or 107 mL (80.5 g; day 110-to-farrow) of the Zn top-dress to feed hoppers once daily for sows assigned to the breed-to-farrow or day 110-to-farrow treatments, respectively (Table 2). The Zn top-dresses alone provided 377 or 8,214 mg of additional Zn per day, respectively, for sows assigned to breed-to-farrow and day 110-to-farrow treatments in addition to the Zn provided by the basal diet. Zinc top-dresses were formulated such that similar total amounts of Zn were fed either for the duration of gestation or directly before farrowing. This approach ensured total amount of Zn provided and timing of the dose were not confounded. The top-dresses used in this study were formulated to provide similar supplemental Zn intake as the highest Zn treatment fed by Holen et al. (2020). Control sows did not receive any top-dressed Zn supplement. Sows received 2.7 kg of feed once daily for the first 30 d of pregnancy then received 2.2 kg once daily until parturition. Throughout gestation, the body condition of sows was assessed by farm staff. Feed allowance was adjusted based on sow body condition score, as sows of ideal body condition received 2.2 kg of feed, thin sows received 2.72 kg of feed and fat sows received 2.0 kg of feed daily. Sows were fed once daily in the morning and water was provided in troughs for the remainder of the day. Upon entry to farrowing rooms, sows were fed 3.0 kg/d of lactation diet for about 5 d until parturition. Immediately after parturition, all sows were fed a common lactation diet and allowed ad libitum access to feed and water.

**Table 1.** Ingredient and nutrient composition of sow diets (as-fed basis)

Ingredient	Gestation	Lactation
Ingredient composition, %		
Corn, coarse ground^1^	31.83	—
Corn, fine ground^1^	31.82	58.04
Soybean meal	2.90	21.60
Choice white grease	—	1.00
DDGS^2^	30.00	15.00
Limestone	1.74	1.41
Monocalcium phosphate, 21% P	0.57	0.92
Salt	0.45	0.45
Lysine HCl	0.25	0.45
dl-Methionine	—	0.08
Tryptophan	0.01	0.03
Threonine	0.01	0.18
Choline—60	0.11	0.10
Dynamate^3^	—	0.38
Defusion 501^4^	0.05	0.10
Ronozyme HP 2700^5^	0.01	0.01
Vitamin-trace mineral premix^6^	0.25	0.25
Total	100.00	100.00
Analyzed nutrient composition		
Moisture, %	13.2	13.4
Crude protein, %	14.3	16.9
Crude fat, %	4.1	4.5
Crude fiber, %	3.1	2.8
Ash, %	5.1	5.7
Calcium, %	0.92	1.02
Phosphorus, %	0.60	0.63
Zinc total, mg/kg	199.7	332.2

^1^Coarse ground corn was milled to a particle size of 900 to 1,000 microns and fine ground corn was milled to 550 to 600 microns.

^2^Corn Distiller’s dried grains with solubles.

^3^Dynamate: feed grade potassium and magnesium sulfate (The Mosaic Company, Plymouth, MN).

^4^Defusion 501: anti-caking flow agent (Promote, Lewisburg, OH).

^5^HiPhos 2,700 (DSM Nutritional Products, Parsippany, NJ) provided an estimated release of 0.10% STTD P.

^6^Contained the following nutrients per kg of premix: Vitamin A, 3,960,000 IU (Vitamin A acetate); Vitamin D_3_, 792,000 IU (cholecalciferol); Vitamin E, 26,400 IU (d1-α-tocphorol acetate); menadione, 1,760 mg (menadione nicotinamide bisulfite); riboflavin, 3,960 mg (crystalline riboflavin); niacin, 19,800 mg (Nicotinamide); pantothenic acid, 13,200 mg (d-Calcium Pantothenate); pyridoxine, 1,320 mg (Pyridoxine Hydrochloride); Vitamin B_12_, 15 mg (Cyanocobalamin); folic acid, 528 mg (Folic acid); biotin, 88 mg (Biotin); thiamin, 880 mg (Thiamin Mononitrate); phytase, 264,000 FTU (Axtra PHY TPT, Danisco Animal Nutrition, Marlborough, UK); zinc, 110,000 mg/kg (Zinc Hydroxychloride, IntelliBond zinc, Micronutrients, Indianapolis, IN); iron, 88,000 mg/kg (Ferrous sulfate); manganese, 44,000 mg/kg (Manganous oxide); chromium, 176 mg/kg (KEMTrace Chromium propionate, Kemin Industries, Inc., Des Moines, IA); copper, 17,600 mg/kg (Basic copper chloride); iodine, 308 mg/kg; selenium, 264 mg/kg (132 mg/kg Sodium selenite and 132 mg/kg Sel-Plex; Alltech, Lexington, Kentucky).

**Table 2.** Ingredient and zinc composition of top-dresses (as-fed basis)

Ingredient,%	Breed-to-farrow top-dress	Day 110-to-farrow top-dress
Corn	96.8	71.4
Choice white grease	1.00	1.00
Zinc sulfate, monohydrate^1^	2.20	27.6
Total	100.0	100.0
Analyzed zinc concentration, mg/kg	8,387	102,033

^1^Minimum of 35.5% zinc.

Sows were housed individually in stalls during gestation on partially slatted concrete floors. Stalls were located in a mechanically ventilated, heated gestation barn. Feed for sows was dropped once daily into a common trough at the front of each stall that was connected to adjacent stalls. After sows consumed their daily ration, the trough was filled with water for the remainder of the day. Treatments were assigned to a block of gestation stalls to avoid cross-contamination of treatments among adjacent sows. The surface of the feed trough was smooth concrete which allowed sows to completely consume their ration and top-dress before water filled the trough. One “buffer” sow was placed at the end of each treatment block to receive the same dietary treatment as her neighbor but was not included in the experiment. At about day 110 of gestation, sows were moved to individual farrowing stalls within farrowing rooms until weaning of litters. Each farrowing room contained 39 stalls. Farrowing stalls were equipped with one stainless steel feeder and one nipple waterer on a fully slatted floor over a deep manure collection pit. An independent controller within each farrowing room operated all heaters and ventilation fans. One heat lamp was placed in the creep area of each farrowing stall as a supplemental heat source for piglets.

### Sow and Piglet Performance

Sows were identified individually using ear tags. Lameness scores were recorded at initiation of dietary treatments and at about day 110 of gestation. Visual lameness scores were recorded and assigned as sows stood up within stalls according to the following scale: (1) normal: sow standing with weight equally distributed on all feet; or (2) lame: sow with arched back, weight unequally distributed on feet, and difficulty or inability to stand. Body condition scores were recorded at the initiation of treatments, at farrowing, and at weaning using a body condition caliper placed at the last rib of the sow (Knauer and Baitinger, 2015).

Measures of sow reproductive performance included total number of piglets born and total number of pigs weaned per litter. The number of pigs born alive, stillborn, and mummified in each litter were expressed as a percentage of total number of pigs born per litter. Within 12 h of birth and prior to cross-fostering, all piglets were weighed individually and ear tagged (LeeO, PrairiE Systems, Spencer, IA). Ear tags were color-coded to match that of their dam’s assigned dietary treatment. Litter sizes were standardized to about 14 piglets per sow by cross-fostering within 24 h of farrowing. Piglets were fostered within treatment as much as possible; however, this practice was not followed exclusively throughout lactation. Farm staff continued to move pigs after 48 h post-farrowing to further equalize litter size and create fallback litters of small pigs. All piglets were processed according to the standard operating procedure established by the farm within 72 h of birth. Piglet processing included tail docking, administering injectable iron (150 mg, Uniferon 200, Pharmacosmos, Inc., Watchung, NJ), and castration of male piglets. The incidence of stillborn and mummified piglets were recorded at birth and birth weight for stillborn pigs was recorded. Stillborn and mummified pigs were included in the count of total pigs born per sow. Piglet survivability was calculated as the number of pigs weaned per litter divided by the number of pigs born alive per litter and included any cross-fostered pigs. Piglets were monitored daily for instances of morbidity and mortality. Any pigs that died during the study were weighed and the date of death, age, sex, and piglet ear tag identification were recorded in the LeeO database. One day before weaning, pigs were weighed and pig inventory was recorded. Piglets were weaned at about 19 ± 6 d of age.

### Sample Analysis

Two random samples of Zn top-dresses, gestation, and lactation diets were collected at the initiation of the experiment and throughout the feeding of dietary treatments to each farrowing group. All samples were stored at −20°C until shipment for analysis. Diet and top-dress samples were sent to Minnesota Valley Testing Laboratories, Inc. (New Ulm, MN) for proximate analysis and determination of zinc concentration. Standard procedures (AOAC, 2006) were followed for analysis of moisture (method 930.15), ash (method 942.05), fat (method 2003.05), crude fiber (method BA6A-05), crude protein (method 990.03), calcium (method 985.01), phosphorus (method 985.01), and zinc (method 985.01) concentrations.

### Statistical Analysis

Experimental data were analyzed using the PROC GLIMMIX procedure of SAS (version 9.4, SAS Institute, Inc., Cary, NC) with a Gaussian distribution. Sow was considered the experimental unit. The statistical model considered the fixed effect of dietary treatment and the random effect of farrowing group (cohorts of sows that farrowed within 1 wk). Other random effects such as sow parity and farrowing room were tested as random effects but removed from the final model because they did not explain a significant portion of variation in response criteria. Pigs were categorized into low (<1.00 kg), normal (1.01 to 1.75 kg), and heavy (>1.76 kg) birth weight categories. The threshold of 1.0 kg was selected to define low birth weight pigs, as piglets weighing about 1.0 kg at birth experience greater mortality rates before weaning compared to pigs born with heavier weights (Calderón Díaz et al., 2017; Feldpausch et al., 2019; Zeng et al., 2019; Holen et al., 2020). Piglet growth data were analyzed using a statistical model that contained fixed effects of dietary treatment and farrowing group with their interaction and pig nested within sow as random terms. Treatment means were separated using the PDIFF option with the Tukey-Kramer adjustment for multiple comparisons. A binomial distribution was used to evaluate percentage of sows remated, piglet survival to weaning, and litter traits (pigs born alive, stillborn pigs, mummified pigs, and pigs weaned per litter). Time was included in the analysis of sow caliper data due to the repeated nature of these data. All data are reported as least square means and considered statistically significant at *P* < 0.05 with *P* < 0.10 considered a trend.

## Results and Discussion

### Chemical Analysis

Zinc concentration of the gestation and lactation diets were slightly higher than formulated values, while the zinc concentration of the breed-to-farrow top-dress was slightly lower than expected and the day 110-to-farrow top-dress was higher than formulated. Based on the analyzed Zn concentration of the gestation diet and the top-dresses, sows fed the control diet consumed 56,425 mg of total Zn (206 mg/kg dietary Zn), sows fed the breed-to-farrow top-dress received 97,940 mg of Zn (353 mg/kg dietary Zn), and sows fed the day 110-to-farrow top-dress received 97,474 mg Zn when the entirety of gestation was considered. Sows assigned to the day 110-to-farrow treatment consumed a diet that contained 206 mg/kg through day 109 of gestation and then consumed 4,285 mg/kg (diet and top-dress) from day 110 to farrowing.

### Sow Performance

No differences were observed for sow parity or gestation length (Table 3). Caliper scores at day 5 of gestation, farrowing, and weaning differed based on dietary Zn treatment (*P* = 0.007). However, the magnitude of change in caliper scores from farrowing to weaning were not different regardless of dietary treatment. Additionally, the incidence of lameness of sows at the allotment or at day 110 of gestation was not different among treatments (data not shown). Lactation length was slightly longer for sows fed the control diet compared to sows fed the breed-to-farrow top-dress (*P* = 0.001). However, this small difference likely had little biological significance and was likely a random difference unrelated to dietary treatments. The days to first service after weaning and percentage of sows mated did not differ among treatments. The percentage of sows rebred was lower than typical industry standards due to the farm’s specific culling procedure, the introduction of new gilts for breeding, and a change in health status of the herd.

**Table 3.** Effect of timing of supplemental zinc feeding to gestating sows on farrowing performance

Item	Control^1^	Breed-to-farrow^2^	Day 110-to-farrow^3^	SEM	*P*-value
No. of sows and litters	92	82	93	—	—
No. of pigs	1,375	1,257	1,358	—	—
Parity	3.1	3.0	3.0	0.16	0.892
Caliper score:^4^					
day 5 of gestation^4^	15.7	15.1	15.5	0.24	Treatment = 0.007 Treatment*time = 0.565
Farrow^4^	15.9	15.5	15.2		
Wean^4^	14.5	13.9	13.7		
Change	−1.5	−1.6	−1.5	0.29	0.897
Gestation length, d	115.6	115.5	115.7	0.11	0.309
Lactation length, d	19.5^a^	19.0^b^	19.3^ab^	0.09	0.001
Days to service	5.5	5.3	5.1	0.45	0.801
Sows mated^5^, %	66.7	70.8	68.4	5.00	0.804
Total born/litter	16.5	16.2	16.3	0.37	0.873
Born alive^6^, %	90.9^b^	93.2^a^	90.6^b^	0.22	<0.001
Stillborn^6^, %	7.5^a^	4.8^c^	6.9^b^	0.61	<0.001
Mummified^6^, %	1.5^c^	2.0^b^	2.4^a^	0.12	<0.001
Foster on/litter	2.2	1.8	2.0	0.43	0.844
Foster off/litter	2.2	2.0	2.0	0.31	0.881
Missing/litter	1.6	1.2	1.3	0.23	0.407
Pigs weaned/litter	11.8	12.2	12.0	0.38	0.848
Survivability^7^, %	72.3^c^	74.5^a^	73.8^b^	2.3	<0.001
Litter stillborn weight, kg	2.5^xy^	2.1^x^	2.8^y^	0.20	0.073
Litter birth weight, kg	22.6	22.7	21.5	0.48	0.141
Litter ADG^8^, kg	2.3	2.4	2.4	0.08	0.476
Litter wean weight, kg	65.9	70.0	68.2	1.76	0.267

^abc^Means within a row with different superscripts differ (*P* < 0.05).

^xy^Means within a row with different superscripts tend to differ (*P *< 0.10).

^1^Diet containing 206 mg/kg supplemental Zn as zinc hydroxychloride (IntelliBond Zinc, Micronutrients, Indianapolis, IN).

^2^Diet containing 353 mg/kg supplemental Zn as Control + ZnSO4·H_2_O fed from day 5 after breeding until farrowing.

^3^The Control diet (206 mg/kg) was fed until day 109 of gestation then a diet containing 4,285 mg/kg supplemental Zn as Control + ZnSO_4_·H_2_O was fed from day 110 of gestation to farrowing.

^4^Body condition scores were evaluated at the last rib using a sow caliper. Data were analyzed considering the repeated observations over time.

^5^Calculated as: (number of sows mated within 7 d of weaning/total sows at weaning) × 100.

^6^Percent of total born.

^7^Calculated as: (number of pigs weaned per sow/number of pigs born alive per sow) × 100.

^8^ADG, average daily gain.

The number of total pigs born per litter was not influenced by dietary treatment. However, breed-to-farrow sows had an increased percentage of pigs born alive compared to sows fed the control diet or to sows fed the day 110-to-Farrow top-dress (*P *< 0.001). Additionally, the percentage of stillborn pigs was lower (*P *< 0.001) for breed-to-farrow sows compared to the control and day 110-to-farrow sows. These results agree with those of Vallet et al. (2014) who observed a reduced frequency of stillborn pigs per litter with increasing dietary Zn (453 mg/kg) fed to pregnant gilts. The authors proposed that supplemental Zn could increase carbonic anhydrase activity, a metalloenzyme involved in carbon dioxide regulation, allowing piglets to tolerate elevated CO_2_ levels during farrowing. Piglets born in large litters and pigs born later in the birth order are more likely to experience asphyxia due to the effects of prolonged uterine contractions and die (Alonso-Spilsbury et al., 2007). Potentially, elevated Zn consumed by breed-to-farrow sows made pigs more robust in utero and prepared piglets more adequately than piglets of control and day 110-to-farrow sows to survive the rigors of parturition. This may explain the increase in percentage of pigs born alive and decreased percentage of stillborn pigs in the breed-to-farrow treatment.

Zinc is a cofactor in the antioxidant enzyme, (Cu/Zn) superoxide dismutase, which is responsible for converting superoxide to oxygen and hydrogen peroxide. High levels of Zn may reduce oxidative stress during pregnancy (Mistry and Williams, 2011). Cony et al. (2023) observed reduced presence of meconium in piglets at birth when they were fed high Zn (625 mg/kg) throughout gestation, suggesting reduced maternal-fetal stress during farrowing. Udomchanya et al. (2019) observed long farrowing periods increase incidence of stillborn pigs, such that the number of stillborn pigs steadily increases for sows with farrowing durations longer than 4 hours (Gourley et al., 2020). Feeding high Zn levels throughout gestation in the current study may have prompted shorter farrowing durations such that incidence of stillborn pigs was reduced, but farrowing duration was not measured. Elevated levels of dietary Zn fed during gestation may alleviate the increase in stillborn pigs often observed in large litter sizes (Vanderhaeghe et al., 2010). Kalinowski and Chavez (1984) observed sows experienced prolonged farrowing duration when fed 13 mg/kg compared to 63 mg/kg of Zn in the last 4 wk of gestation. Similar results were obtained when pregnant gilts were fed Zn-deficient diets in the last third of gestation and experienced long farrowing durations and decreased viability of piglets at parturition (Palludan and Wegger, 1976). We acknowledge that supra-supplementation of Zn may not elicit effects similar to the correction of a Zn deficient state; however, zinc (Zn^2+^) ions may interact with other ions such as calcium (Ca^2+^) and magnesium (Mg^2+^) to aid in muscle contraction through enhanced cell-to-cell signaling (Maret, 2017). Richardson and Drake (1979) fed a high Zn diet to rats and observed decreased fatigue of striated muscle compared to rats fed a control diet. Similar findings have been observed in humans where high Zn increased muscle strength during exercise (Krotkiewski et al., 1982). These researchers evaluated the performance of striated skeletal muscle, whereas the uterus is predominately smooth muscle and may respond differently to high dietary levels of Zn. Perhaps in the present study, high Zn aided in uterine contractions and decreased muscle fatigue, allowing for faster and more efficient expulsion of fetuses compared with sows fed the control diet. Further investigation is required to evaluate if high levels of Zn decrease the duration of farrowing, as contradictory results have been presented by Vallet et al. (2014) who observed high Zn (453 mg/kg) increased birth intervals in early parturition but reduced the frequency of stillborns. Cony et al. (2023) observed no reduction in farrowing duration when 625 mg/kg of Zn was fed throughout gestation or starting at day 80 to gestating sows. Stillborn pigs per litter born to breed-to-farrow sows in the present study tended to weigh less (*P *= 0.073) compared to stillborn pigs born to day 110-to-farrow sows. The percentage of mummified pigs was increased for day 110-to-farrow sows compared to control and breed-to-farrow sows, while breed-to-farrow sows experienced a 0.5% increase in mummified pigs compared to control sows (*P* < 0.001).

Feeding minerals above the stated NRC (2012) requirement may result in biological signs of toxicity. In the present study, sows fed either top-dress showed no signs of gastrointestinal upset, reduced feed intake, lameness, or reduced reproductive performance, and all sows consumed their allotted top-dress daily. Hill et al. (1983) determined the effects of long-term high Zn feeding on gestating swine. The authors fed gilts 0, 50, 500, or 5,000 mg/kg of dietary Zn as zinc oxide from 30 kg BW over two parities and observed slight reductions in reproductive performance for sows fed 5,000 mg/kg of Zn in the diet. From the results of hill and coworkers, we were confident the levels of Zn fed in the present study did not depress sow performance.

### Piglet Survival

Pigs were cross-fostered initially within 24 h of farrowing; however, additional fostering events occurred throughout the lactation period to further equalize litter size. Piglets were fostered to other litters within the same treatment as much as possible but there were some piglets that were fostered to litters of a different treatment. For control, breed-to-farrow, and day 110-to-farrow litters, 8.5%, 6.9%, and 5.6% of piglets born alive, respectively, were fostered to treatments different than their birth dam. There were no differences between the total number of pigs fostered on sows or off sows regardless of dietary treatment (Table 3). Over the duration of the study, 58 pigs that were present at birth were not present at weaning and their survivability outcome could not be determined. Presumably, these pigs died or were fostered to litters not a part of the experiment and were not recorded on barn records; however, they were included in statistical analysis to determine any treatment bias in missing pigs. We observed no differences in the number of pigs per litter that were missing across treatments. The number of pigs weaned per litter did not differ due to dietary Zn treatment. Missing pigs were included in the calculation of piglet survival and were considered as dead pigs. Piglet survivability ranged from 74.5% to 72.3% and was increased for pigs born to breed-to-farrow sows compared to day 110-to-farrow and control sows (*P* < 0.001), as control sows experienced the lowest piglet survivability (Table 3).

No differences in overall piglet mortality were observed regardless of dietary treatment (Table 4). These results conflict with Holen et al. (2020) who observed a tendency for decreased overall mortality (15.0% to 12.2%) for pigs born to sows fed 595 mg/kg of Zn starting on day 85 of gestation. Cony et al. (2023) fed 625 mg/kg of Zn throughout gestation or 625 mg/kg starting on day 80 and observed piglet mortality to decrease for pigs born to sows fed either Zn treatments compared to sows fed 206 mg/kg of Zn. Mortality in the study reported by Cony et al. (2023) was exceptionally low (4.6%; control vs. 4.2%; high Zn treatments). Interestingly, Hu et al. (2023) fed gestating sows 360 mg/kg supplemental Zn 4 d before farrowing throughout lactation and observed decreased piglet mortality for sows fed high Zn compared to sows fed 120 mg/kg of Zn. Moeller et al. (2022) fed 957 mg/kg of ZnSO_4_ to sows starting on day 84 of gestation and observed no reduction in mortality, although noted that lack of a beneficial effect could have been attributed to the unstable PRRS status of the farm. Results of these studies and the present study concur that high levels of dietary Zn influence post-partum or intrapartum mortality of piglets. The timing of Zn supplementation and Zn dosage differs across studies; thus, the optimal timing and amount of dietary Zn need to be validated. It appears that high levels of dietary Zn fed to gestating sows have a positive impact on their piglets. However, more research is warranted to establish an optimal Zn supplementation protocol that limits unnecessary fecal Zn excretion.

**Table 4.** Effect of timing of supplemental zinc feeding to gestating sows on preweaning mortality of pigs by birth weight category

Item	Control^1^	Breed-to-farrow^2^	Day 110-to-farrow^3^	SEM	*P*-value
Overall:					
Born alive	1,375	1,257	1,358	—	—
Stillborn	116	66	105	—	—
Mummified	23	27	37	—	—
Missing pigs	18	14	26	—	—
Pigs born alive^4^, %	90.9^b^	93.1^a^	90.6^b^	0.73	0.003
Missing^5^, %	1.1	0.9	1.6	0.29	0.200
Pigs weaned^6^, %	82.0	83.2	82.4	1.05	0.727
Mortality^7^, %	17.8	16.7	17.3	1.04	0.743
Low birth weight (≤1.00 kg):					
Total pigs^8^	140	144	173	—	—
Born alive^4^, %	84.3	91.0	91.1	2.51	0.108
Missing^5^, %	7.0	3.4	4.7	2.77	0.403
Pigs weaned^6^, %	38.5	36.5	44.5	4.28	0.351
Mortality^7^, %	56.2	60.9	52.0	5.04	0.305
Incidence of low birth weight^6^, %	12.3^b^	15.1^ab^	18.1^a^	0.98	<0.001
Normal birth weight (1.01 to 1.75 kg):					
Total pigs^8^	947	822	972	—	—
Born alive^4^, %	92.1^b^	95.1^a^	92.5^b^	1.11	0.027
Missing^5^, %	0.85	0.88	1.54	0.47	0.259
Pigs weaned^6^, %	81.9^y^	85.3^xy^	85.5^x^	1.43	0.074
Mortality^7^, %	17.9^x^	14.7^xy^	13.8^y^	1.48	0.068
Incidence of normal birth weight^6^, %	69.2	67.4	68.1	1.28	0.619
Heavy birth weight (≥1.76 kg):					
Total pigs^8^	401	355	299	—	—
Born alive^4^, %	96.3	97.0	96.1	1.08	0.805
Missing^5^, %	0.26	0.29	0.35	0.30	0.978
Pigs weaned^6^, %	94.0	95.0	93.7	1.28	0.745
Mortality^7^, %	6.0	4.9	6.3	1.28	0.745
Incidence of heavy birth weight^6^, %	18.0^a^	16.9^a^	13.2^b^	1.01	0.002

^abc^Means within a row with different superscripts differ (*P* < 0.05).

^xy^Means within a row with different superscripts tend to differ (*P *< 0.10).

^1^Diet containing 206 mg/kg supplemental Zn as zinc hydroxychloride (IntelliBond Zinc, Micronutrients, Indianapolis, IN).

^2^Diet containing 353 mg/kg supplemental Zn as Control + ZnSO_4_·H_2_O fed from d 5 after breeding until farrowing.

^3^The Control diet (206 mg/kg) was fed until day 109 of gestation then a diet containing 4,285 mg/kg supplemental Zn as Control + ZnSO_4_·H_2_O was fed from day 110 of gestation to farrowing.

^4^Percent of total born.

^5^Calculated as: pigs that were expected to be weaned but weaning weight or outcome was unknown/number of pigs born alive.

^6^Percent of born alive.

^7^Calculated as: pigs that died from birth to weaning/number of pigs born alive.

^8^Total born within each birth weight category is the summation of born alive and stillborn pigs.

We hypothesized that supplementing high levels of Zn to gestating sows would decrease preweaning mortality of low birth weight pigs similar to the results of Vallet et al. (2014) and Holen et al. (2020). However, mortality of pigs weighing less than 1.0 kg was not affected by high Zn treatments fed to sows in this study. In these previous studies, gilts or sows were fed 453 to 595 mg/kg of total supplemental Zn starting at days 80 or 85 of gestation until farrowing, respectively. No evidence for differences in the mortality of heavy birth weight pigs (≥1.76 kg) were observed; however, a trend for decreasing mortality (*P *= 0.068) of normal birth weight pigs (1.0 to 1.75 kg) was observed in litters farrowed by day 110-to-farrow sows compared with control sows and breed-to-farrow sows intermediate. An increased percentage of low birth weight pigs were born to sows fed the day 110-to-farrow top-dress compared to pigs born to control sows (*P *< 0.001) with the breed-to-farrow pigs intermediate. Additionally, a reduced percentage of heavy birth weight pigs were observed for day 110-to-farrow sows compared to control and breed-to-farrow sows. No differences were observed in the percentage of low or heavy-birth-weight pigs born alive or weaned. Interestingly, a higher percentage of normal birth weight pigs were born alive to sows fed 353 mg/kg of total supplemental Zn throughout gestation compared with control sows and day 110-to-farrow (95.1% vs. 92.1 and 92.5%; *P *= 0.027). These results suggest normal birth weight pigs born to sows fed the breed-to-farrow top-dress were more robust during parturition and more likely to survive the farrowing process compared to similar-sized pigs born to control sows. Furthermore, more normal birth weight pigs tended to be weaned from sows fed elevated Zn in the 5 d before parturition compared to pigs born to sows fed the control diet.

The same facility location was used in the present study as was used by Holen et al. (2020). The overall total amount of Zn supplemented during gestation in the present study was designed to be similar to the high Zn level (595 mg/kg) fed by Holen et al. (2020) which decreased preweaning mortality of low birth weight pigs in their study. Vallet et al. (2014) fed 453 mg/kg of supplemental Zn and observed a reduction in mortality of pigs with low birth weight. Unlike the present study, the supplemental Zn was fed starting on days 80 or 85 of gestation. No response in piglet mortality was observed in our study when high Zn was fed throughout gestation or the last 5 d of gestation. This difference in the supplementation period suggests that timing of Zn supplementation is important. Cony et al. (2023) fed 625 mg/kg of supplemental Zn throughout gestation or starting at day 80 of gestation and observed overall preweaning mortality to decrease for both high Zn diets compared to sows fed a standard level of Zn. Therefore, one may conclude that the optimal level of dietary Zn is 625 mg/kg which is higher than the 353 mg/kg fed in the present study. Interestingly, feeding 625 mg/kg Zn in late gestation elicited reductions in piglet mortality in two different studies (Holen et al., 2020; Cony et al., 2023). Thus, high Zn diets should be fed for a short duration such as only in late gestation to minimize unnecessary Zn excretion. Rapid fetal growth begins around day 70 of gestation (McPherson et al., 2004). Our results combined with recently published studies suggest that feeding high Zn concentrations (595 to 625 mg/kg) during late gestation (beginning about d 80) is key in modulating fetal development to decrease preweaning mortality. A limitation of our current study is that a treatment diet starting at day 85 of gestation was not included. Inclusion of this additional treatment would have allowed a direct comparison of late gestation with supplementation throughout gestation. Furthermore, the lack of Zn-induced reductions in preweaning mortality of piglets in the present study may be attributed to a heavier average birth weight compared to that observed by Holen et al. (2020), as heavier birth weight is correlated positively with preweaning survival of piglets (Zeng et al., 2019).

### Piglet Performance

Birth weight is highly associated with preweaning mortality (Feldpausch et al., 2019) and is often regarded as the greatest predictor of survivability. In the current study, the pig population was categorized into three birth weight classifications (low, normal, or heavy). Overall piglet birth weight tended to be heavier for pigs born to control pigs compared to day 110-to-farrow pigs with breed-to-farrow pigs intermediate (*P* = 0.054; Table 5). However, no evidence for differences were observed for piglet birth weight in any of the three birth weight classifications across dietary treatments (Table 5). Weaning weight of normal birth weight pigs tended to increase for day 110-to-farrow pigs compared to breed-to-farrow pigs which was driven by increased daily gain experienced by day 110-to-farrow pigs (*P* = 0.007). Litter birth weights, average daily gain of litters, and litter weaning weights did not differ among dietary treatments (Table 3). Results of this study conflict with those of Holen et al. (2020) who observed increased piglet birth weight for pigs born to sows fed 365 mg/kg of total supplemental Zn. Overall piglet birth weight in the present study was 120 g heavier than the overall piglet birth weight observed by Holen et al. (2020). We would expect improved piglet survival with heavier birth weights; however, in the current study, mortality rates were higher than those observed by Holen et al. (2020). Timing of zinc supplementation differed between these two studies and the number of total pigs born per litter was 2.1 pigs higher in the present study. One cannot dismiss the possibility that the higher mortality rate observed resulted from larger litters, perhaps due to genetic line differences. When 360 mg/kg of supplemental Zn from ZnSO_4_ was fed to gestating sows 4 d before farrowing, average piglet birth weight increased compared to sows fed 120 mg/kg of Zn (Hu et al., 2023). Cony et al. (2023) observed piglets born to sows fed a higher amount of supplemental Zn (625 mg/kg) throughout gestation had heavier weaning weights than pigs born to sows fed 206 mg/kg of Zn and a tendency for greater weight gain during the preweaning period. This is in agreement with day 110-to-Farrow piglets of normal birth weight in the present study who experienced increased preweaning growth. Piglet performance was minimally affected in this study unlike the previously mentioned studies; thus, it appears that the timing and dietary Zn concentration fed to gestating sows may be critical. Additional research evaluating the optimal timing of supplementation and dietary level of Zn is needed to elucidate this mineral’s utility as a fetal imprinting tool to enhance post-natal performance of offspring.

**Table 5.** Effect of timing of supplemental zinc feeding to gestating sows on piglet growth performance

Item	Control^1^	Breed-to-farrow^2^	Day 110-to-farrow^3^	SEM	*P*-value
Overall:					
Piglet birth weight, kg^4^	1.56^x^	1.52^xy^	1.47^y^	0.052	0.054
Piglet gain, g/d	205.1	211.8	215.3	5.88	0.130
Piglet weight at weaning, kg	5.66	5.63	5.69	0.287	0.868
Piglet age at weaning, d	19.7^a^	19.0^c^	19.3^b^	0.68	< 0.001
Total piglet gain, g	4,040	4,045	4,161	248.9	0.398
Low birth weight (≤ 1.00 kg):					
Piglet birth weight, kg^4^	0.84	0.82	0.84	0.020	0.536
Piglet gain, g/d	170.9	172.6	171.3	6.21	0.982
Piglet weight at weaning, kg	4.27	4.19	4.23	0.167	0.906
Piglet age at weaning, d	19.9^a^	18.9^c^	19.6^b^	0.70	< 0.001
Total piglet gain, g	3,365	3,288	3,317	160.3	0.919
Normal birth weight (1.01 to 1.75 kg):					
Piglet birth weight, kg^4^	1.44	1.43	1.41	0.015	0.208
Piglet gain, g/d	198.3^b^	206.0^ab^	214.0^a^	4.83	0.007
Piglet weight at weaning, kg	5.36^xy^	5.35^y^	5.57^x^	0.220	0.054
Piglet age at weaning, d	19.6^a^	19.0^c^	19.3^b^	0.71	<0.001
Total piglet gain, g	3,901^b^	3,910^ab^	4,139^a^	212.8	0.026
Heavy birth weight (≥1.76 kg):					
Piglet birth weight, kg^4^	1.95	1.93	1.93	0.022	0.554
Piglet gain, g/d	219.5	228.4	232.3	7.26	0.259
Piglet weight at weaning, kg	6.29	6.31	6.42	0.281	0.701
Piglet age at weaning, d	19.9^a^	19.1^c^	19.3^b^	0.59	<0.001
Total piglet gain, g	4,338	4,377	4,488	265.6	0.620

^abc^Means within a row with different superscripts differ (*P* < 0.05).

^xy^Means within a row with different superscripts tend to differ (*P *< 0.10).

^1^Diet containing 206 mg/kg supplemental Zn as zinc hydroxychloride (IntelliBond Zinc, Micronutrients, Indianapolis, IN).

^2^Diet containing 353 mg/kg supplemental Zn as Control + ZnSO_4_·H_2_O fed from day 5 after breeding until farrowing.

^3^The Control diet (206 mg/kg) was fed until day 109 of gestation then a diet containing 4,285 mg/kg supplemental Zn as Control + ZnSO_4_·H_2_O was fed from day 110 of gestation to farrowing.

^4^Includes pigs that died any time from birth to weaning.

### Subsequent Sow Performance

Subsequent farrowing performance was recorded for sows that were mated after weaning to determine if high Zn supplementation influenced the next reproductive cycle (Table 6). Gestation length was not affected by previous Zn supplementation and the percentage of sows that farrowed who were rebred also did not differ due to previous treatments. The number of pigs born per litter was similar across previous treatments; however, there was evidence for differences in the percentage of pigs born alive as sows previously fed the day 110-to-farrow treatment experienced a reduced percentage of pigs born alive compared to control or breed-to-farrow sows (*P* < 0.001). Interestingly, the number of mummified pigs as a percentage of total born was increased (14.5 vs. 3.2 to 6.3%, *P *= 0.003) for pigs born to sows fed 4,285 mg/kg of supplemental Zn directly before the previous farrowing period compared to breed-to-farrow or control sows. At the conclusion of the trial in May 2021, the farm experienced a porcine reproductive and respiratory syndrome (PRRS) infection. The increased percentage of mummified piglets and decreased percentage of pigs born alive compared with the previous farrowing (Table 3) is likely a result of the PRRS infection. An explanation for the significant differences among Zn treatments is not readily apparent. Similar to the previous farrowing period, there were no differences observed in the number of pigs weaned or mortality of piglets due to dietary treatments fed previously.

**Table 6.** Effect of timing of supplemental zinc feeding to gestating sows on subsequent farrowing performance

Item	Control^1^	Breed-to-farrow^2^	Day 110-to-farrow^3^	SEM	*P*-value
No. sows	42	45	43	—	—
No. pigs	517	568	452	—	—
Parity	3.5	3.7	3.6	0.21	0.784
Gestation length, d	115.7	115.7	116.0	0.22	0.688
Sows farrowed, %	70.0	80.0	68.3	5.73	0.324
Total born/litter	15.0	15.0	14.0	0.62	0.454
Born alive^4^, %	84.4^a^	86.2^a^	76.3^b^	4.83	<0.001
Stillborn^4^, %	8.3	8.9	7.1	1.15	0.480
Mummified^4^, %	6.3^b^	3.2^c^	14.5^a^	3.83	<0.001
Pigs weaned/litter	10.7	10.7	10.0	0.52	0.414
Mortality^5^, %	17.4	14.4	15.3	1.63	0.389

^abc^Means within a row with different superscripts differ (*P* < 0.05).

^1^Diet containing 206 mg/kg supplemental Zn as zinc hydroxychloride (IntelliBond Zinc, Micronutrients, Indianapolis, IN).

^2^Diet containing 353 mg/kg supplemental Zn as Control + ZnSO_4_·H_2_O fed from d 5 after breeding until farrowing.

^3^The Control diet (206 mg/kg) was fed until day 109 of gestation then a diet containing 4,285 mg/kg supplemental Zn as Control + ZnSO_4_·H_2_O was fed from day 110 of gestation to farrowing.

^4^Percent of total born.

^5^Calculated as: pigs that died from birth to weaning/number of pigs born alive.

## Conclusion

In conclusion, preweaning mortality of low birth weight pigs was not reduced by elevated supplemental Zn fed to sows throughout gestation or 5 d before farrowing. However, feeding high levels of Zn throughout gestation reduced fetal mortality and piglet mortality during the farrowing process, as the percentage of pigs born alive increased, and the percentage of stillborn pigs decreased compared to sows fed typical Zn concentrations. The optimal timing of elevated Zn supplementation to improve piglet survival may be around day 85 of gestation when fetal growth. The biological mechanism(s) of zinc’s positive effects on piglet survival is yet to be elucidated.
